# High diversity of nitrifying bacteria and archaea in biofilms from a subsea tunnel

**DOI:** 10.1093/femsec/fiaf032

**Published:** 2025-03-28

**Authors:** Linnea F M Kop, Hanna Koch, Paula Dalcin Martins, Carolina Suarez, Sabina Karačić, Frank Persson, Britt-Marie Wilén, Per Hagelia, Mike S M Jetten, Sebastian Lücker

**Affiliations:** Department of Microbiology, Radboud Institute for Biological and Environmental Sciences, Radboud University, Heyendaalseweg 135, 6525 AJ, Nijmegen, The Netherlands; Department of Microbiology, Radboud Institute for Biological and Environmental Sciences, Radboud University, Heyendaalseweg 135, 6525 AJ, Nijmegen, The Netherlands; Bioresources Unit, Center for Health and Bioresources, AIT Austrian Institute of Technology GmbH, Konrad-Lorenz-Straße 24, 3430 Tulln an der Donau, Austria; Ecosystems and Landscape Dynamics, Institute for Biodiversity and Ecosystem Dynamics, University of Amsterdam, Science Park 904, 1090 GE Amsterdam, The Netherlands; Division of Water Resources Engineering, Faculty of Engineering LTH, Lund University, John Ericssons väg 1, 221 00 Lund, Sweden; Division of Water Environment Technology, Department of Architecture and Civil Engineering, Chalmers University of Technology, Sven Hultins gata 6, 412 96 Gothenburg, Sweden; Institute of Medical Microbiology, Immunology and Parasitology, Universitätsklinikum Bonn, Venusberg – Campus 1, 53127 Bonn, Germany; Division of Water Environment Technology, Department of Architecture and Civil Engineering, Chalmers University of Technology, Sven Hultins gata 6, 412 96 Gothenburg, Sweden; Division of Water Environment Technology, Department of Architecture and Civil Engineering, Chalmers University of Technology, Sven Hultins gata 6, 412 96 Gothenburg, Sweden; Construction Division, The Norwegian Public Roads Administration, Innspurten 11C, 0663 Oslo Norway; Müller-Sars Biological Station, Ørje, PO Box 64, NO-1871 Ørje, Norway; Department of Microbiology, Radboud Institute for Biological and Environmental Sciences, Radboud University, Heyendaalseweg 135, 6525 AJ, Nijmegen, The Netherlands; Department of Microbiology, Radboud Institute for Biological and Environmental Sciences, Radboud University, Heyendaalseweg 135, 6525 AJ, Nijmegen, The Netherlands

**Keywords:** nitrification, microbial diversity, metagenomics, biofilm, concrete deterioration, microbial interactions

## Abstract

Microbial biofilm formation can contribute to the accelerated deterioration of steel-reinforced concrete structures and significantly impact their service life, making it critical to understand the diversity of the biofilm community and prevailing processes in these habitats. Here, we analyzed 16S rRNA gene amplicon and metagenomics sequencing data to study the abundance and diversity of nitrifiers within biofilms on the concrete surface of the Oslofjord subsea road tunnel in Norway. We showed that the abundance of nitrifiers varied greatly in time and space, with a mean abundance of 24.7 ± 15% but a wide range between 1.2% and 61.4%. We hypothesize that niche differentiation allows the coexistence of several nitrifier groups and that their high diversity increases the resilience to fluctuating environmental conditions. Strong correlations were observed between the nitrifying families *Nitrosomonadaceae* and *Nitrospinaceae*, and the iron-oxidizing family *Mariprofundaceae*. Metagenome-assembled genome analyses suggested that early *Mariprofundaceae* colonizers may provide a protected environment for nitrifiers in exchange for nitrogen compounds and vitamin B_12_, but further studies are needed to elucidate the spatial organization of the biofilms and the cooperative and competitive interactions in this environment. Together, this research provides novel insights into the diverse communities of nitrifiers living within biofilms on concrete surfaces and establishes a foundation for future experimental studies of concrete biofilms.

## Teaser

Biofilms on the concrete surface of the Oslofjord subsea road tunnel contain a high diversity of nitrifying microorganisms, which might increase their resilience to fluctuating environmental conditions.

## Introduction

Concrete deterioration and corrosion of steel reinforcements significantly impact the service life of concrete structures (Cámara et al. 2022, Xu et al. 2023). In addition to abiotic processes, microbial colonization can have a strong effect on the degradation of steel-reinforced concrete (Noeiaghaei et al. 2017). Biofilm formation on concrete surfaces, followed by metabolic activities of their microbial inhabitants, can lead to a decrease in pH, facilitating the penetration of substances such as chloride ions, CO_2_, and moisture into the concrete–steel interface (Noeiaghaei et al. 2017). The involvement of sulfur-cycling bacteria in the biodeterioration of concrete sewer systems and petrochemical pipelines is well-documented (Little et al. 2000, Satoh et al. 2009, Wu et al. 2020). In contrast, our understanding of biodeterioration processes in marine environments is limited. Recent 16S rRNA gene-based analyses of microbial communities in biofilms formed on steel fiber-reinforced sprayed concrete surface of a subsea road tunnel in the Oslofjord (Norway) indicated that these systems were not dominated by sulfur-cycling microorganisms. Instead, autotrophic nitrogen- and iron-oxidizing microorganisms were found in high abundances, along with heterotrophic bacteria, similar to the microbial community found in the corresponding Oslofjord sediments (Karačić et al. 2018, 2024). In addition, shotgun metagenomic sequencing revealed the presence of putative novel nitrogen-transforming members of the phylum *Plantomycetota* inhabiting these biofilms (Suarez et al. 2022, 2023). The high abundance of nitrogen-cycling microorganisms in this subsea environment is likely due to a higher concentration of ammonium in the water leaking into the tunnel relative to seawater. The source of ammonium is likely the remineralization of organic matter in the sediment overlying the tunnel (Suarez et al. 2023). In addition, under acidic conditions, ammonium can be formed from abiotic reactions between nitrate and metals (4 Fe^0^ + NO_3_^−^ + 10 H^+^ = 4 Fe^2+^ + NH_4_^+^ + 3 H_2_O), and thus might be continuously regenerated in water leaking through the tunnel wall (Zhang et al. 2009). Interestingly, the biofilms contained relatively high concentrations of iron and manganese (Hagelia 2007, 2011, Karačić et al. 2018), and diverse communities of nitrifiers have often been observed at high abundances in sediments with Fe–Mn deposits, where they are primary producers (Nitahara et al. 2011, 2017, Kato et al. 2019, Molari et al. 2020, Hollingsworth et al. 2021). However, the reason for such high nitrifier diversity in these ecosystems and their potential link to iron or manganese cycling remains to be elucidated.

Nitrifiers have previously been implicated in the degradation of concrete in a cooling tower (Kaltwasser 1976), where the nitric acid (HNO_3_) produced by ammonia and subsequent nitrite oxidation was thought to be the cause of concrete deterioration. Such biodeterioration caused by nitrifiers due to acidification has also been observed on concrete in wastewater treatment plants (Leemann et al. 2010) and natural stone (Meincke et al. 1989, Baumgärtner et al. 1991, Sand and Bock 1991). Similarly, the biofilm-induced acidification from pH 7.5–8 to 5.5–6.5 in the seeping saline water led to the disintegration of the cement paste matrix in the Oslofjord tunnel (Hagelia 2011, Karačić et al. 2018). Contrastingly, at more alkalic conditions, a decrease in pH from about 9 to 8 by nitrifiers does not significantly increase concrete corrosion (Zhang et al. 2010), and a direct causal relationship between nitrification in tunnel biofilms and increased corrosion has yet to be established.

Microbial biofilms, such as the ones that deteriorate man-made structures, are generally composed of complex microbial communities adhering to surfaces by producing extracellular polymeric substances (EPS). Growing in biofilms provides numerous advantages, such as physical protection and specialized niches formed by gradients of nutrients, waste products, and signaling compounds (Stewart and Franklin 2008, Antunes et al. 2019). The heterogeneity of microenvironments contributes to increased microbial diversity within the biofilm (Flemming et al. 2016, Zhang et al. 2019). In addition, biofilms protect against desiccation and increase adsorption of nutrients and other molecules to the biofilm matrix (Flemming et al. 2016). Pores and channels within biofilms can facilitate liquid transport at higher rates and over greater distances than diffusion would allow (Wilking et al. 2013). While certain conditions may promote competitive interactions, biofilms are thought to be habitats where cooperative interactions between microorganisms—for example through the exchange of metabolites such as amino acids—are favorable (Ren et al. 2015, Zelezniak et al. 2015).

As niche differentiation in nitrifying assemblages is likely controlled by factors such as substrate concentrations (Maixner et al. 2006, Gruber-Dorninger et al. 2015), gradients within biofilms may provide conditions suitable for differentially adapted populations, thereby stimulating biodiversity. In addition, nitrifiers growing in biofilms may adapt to suboptimal pH ranges (Keen and Prosser 1987, Allison and Prosser 1993, Tarre and Green 2004) and are more resistant to starvation (Batchelor et al. 1997) and inhibitors (Powell and Prosser 1991). A possible explanation for the enhanced growth of nitrifiers in biofilms could be the adsorption of ammonium to the anionic components of EPS (Nielsen 1996). At low substrate concentrations, its subsequent gradual release might help to maintain cellular activity. Furthermore, the accumulation of quorum-sensing molecules was shown to be involved in reducing the lag phase in *Nitrosomonas europaea* cultures when growing in biofilms compared to suspended growth (Batchelor et al. 1997).

Notably, Karačić et al. (2018, 2024) showed that nitrifiers were not only highly abundant but also highly diverse in the tunnel biofilms. Intrigued by the unusual cooccurrence of so many nitrifying groups in one habitat, we reanalyzed existing and new 16S rRNA gene amplicon and metagenomic sequencing data to further explore nitrifier biodiversity. We hypothesize that niche differentiation and metabolic flexibility allow a high number of different nitrifiers to coexist within the biofilm matrices, which contributes to community resilience. In addition, we identified microbial groups correlating with nitrifiers and discussed their putative metabolic links to them.

## Methods

### Sampling

The Oslofjord subsea tunnel is located under the Oslofjord near Drøbak, Norway (59.66 472 N, 10.61306 E). Steel fiber-reinforced sprayed concrete, applied directly on the rock mass, is used as a final support together with rock bolts. Biofilm growth thrives on the highly bioreceptive, rough, and tortuous surfaces of sprayed concrete, especially in areas where cracks in both the ambient rock mass and the concrete layer allow infiltration of seawater-like saline groundwater (Hagelia 2011). Details on sampling locations and procedures were published previously by Karačić et al. (2018, 2024) and Suarez et al. (2022). In short, for amplicon sequencing, samples (*n* = 95) were collected during four different years (2015, 2016, 2017, and 2019) from several sites within three different locations, designated pump station (P), test site (T), and main tunnel (M). For metagenomics, only samples from the years 2016, 2017, 2019, and 2020 from sites P and T were sequenced (*n* = 8). Concrete was sprayed and biofilms developed at locations P and M from 1999 onward, whereas concrete at location T was only sprayed in 2010 and biofilm started to form in 2013 due to increasing water flow.

### 16S rRNA gene analyses

In this study, we reanalyzed 16S rRNA gene Illumina MiSeq sequencing data of the V4 region published by Karačić et al. (2018, 2024) (Bioproject accession numbers PRJNA481470 and PRJNA1061464). Additional samples from location M taken in the years 2017 and 2019 were sequenced as previously described (Karačić et al. 2018, 2024) and are also available under the accession number PRJNA1061464. While the samples from 2015 and 2016 were sequenced with MiSeq reagents V2, all samples from 2017 and 2019 were sequenced with MiSeq reagents V3. DADA2 v1.24.0 was used to process sequences and infer amplicon sequence variants (ASVs) (Callahan et al. 2016). Since error rates might differ between the sequencing runs, samples from the 2015/2016 and the 2017/2019 datasets were analyzed separately during denoising and then merged prior to the chimera removal step. Taxonomy was assigned using the GTDB r214 (Parks et al. 2020). For simplicity, we use Genome Taxonomy Database Toolkit (GTDB) placeholder names to refer to family and genus-level lineages without cultured representatives throughout the manuscript.

ASVs were analyzed in R v4.2.2 (R Core Team 2022) using the R package mia v1.6.0 (Ernst et al. 2022). Linear relationships between the families of ASVs were estimated using SECOM (Lin et al. 2022) with the ANCOMBC R package using the secom_linear function (Lin and Peddada 2020, Lin et al. 2022) with the Pearson correlation coefficient and obtaining the sparse correlation matrix obtained by thresholding. Only families that cooccurred in at least half of the samples were included in the analyses.

### Metagenome sequencing and analyses

In this study, we reanalyzed metagenomic sequencing data of eight samples from the P and T sites from four different years (2016, 2017, 2019, and 2020; Bioproject accession number PRJNA755678) published by Suarez et al. (2022). For this, metagenome-assembled genomes (MAGs) from the coassembly of all eight samples were classified using the GTDB Toolkit (v2.1.1) classification workflow (classify_wf) with the r214 reference database (Chaumeil et al. 2019). GTDB placeholder names are used throughout the manuscript to refer to family and genus-level lineages without cultured representatives. Genome completeness and redundancy were assessed using CheckM (v1.0.11) (Parks et al. 2015).

MAGs were annotated using DRAM (Shaffer et al. 2020) with prodigal gene calling (Hyatt et al. 2010). The KOfam (Aramaki et al. 2020), UniRef90 (Suzek et al. 2015), Pfam (El-Gebali et al. 2019), and dbCAN (Zhang et al. 2018) databases were used for annotation. These annotations were combined with results obtained from MicrobeAnnotator using Diamond as the search tool (Buchfink et al. 2014, Ruiz-Perez et al. 2021). In addition, key genes were manually curated using blastp searches of representative proteins (e-value ≤0.00001, bitscore ≥30, % identity ≥30%, and query cover ≥80). Combined annotations can be found in Table S1. Putative [NiFe] hydrogenases were identified in the annotation based on Pfam accession PF00374 and classified using HydDB (Søndergaard et al. 2016). Marker proteins for iron metabolism were searched with FeGenie (Garber et al. 2020). CRISPR arrays and Cas proteins were identified using the online CRISPRCasFinder (https://crisprcas.i2bc.paris-saclay.fr/) with default settings (Couvin et al. 2018).

### Phylogenetic analyses

For calculating phylogenomic trees with the Oslofjord biofilm MAGs, genomes of the class *Nitrospinia*, the order *Nitrospirales*, and the genera *Nitrosomonas* and *Nitrosopumilus* were downloaded from NCBI (August 2023). Additional *Nitrospinota* genomes were downloaded from IMG and added to the OceanDNA dataset (Nishimura and Yoshizawa 2022). All downloaded genomes were classified using the GTDB-Tk (v2.1.1) classification workflow (classify_wf) with the r214 reference database (Chaumeil et al. 2019). Genomes of the genera *Nitrosomonas* and *Nitrosopumilus*, the family UBA8639 and the class *Nitrospinia* were retained. Genome completeness and redundancy were assessed using CheckM (v1.0.11) (Parks et al. 2015) and nonredundant genomes were selected using dRep with an average nucleotide identity (ANI) cutoff ≥99%, the “average” clustering algorithm, and “ANImf” for secondary clustering (v2.4.2) (Olm et al. 2017). For all subsequent analyses, the nonredundant reference genomes were combined with the Oslofjord biofilm MAGs. For the bacterial genomes (family UBA8639, class *Nitrospinia*, and genus *Nitrosomonas*), concatenated alignments of 92 extracted core genes were constructed using the MAFFT aligner implemented in the UBCG pipeline (v3.0) (Katoh 2002, Na et al. 2018). For the *Nitrosopumilus* genomes, the anvi′o (v. 7.1) function “anvi-get-sequences-for-hmm-hits” was used to extract 36 archaeal ribosomal genes for the concatenated alignment constructed using the muscle aligner (v.3.8.1551) implemented in anvi′o (Edgar 2004, Eren et al. 2020).

IQ-Tree (v1.6.12) ModelFinder identified GTR+F+I+G4 as the best fitting model for the *Nitrosomonas* and UBA8639 datasets, SYM+I+G4 for *Nitrospinia*, and JTTDCMut+F+I+G4 for the *Nitrosopumilus* gene set (Kalyaanamoorthy et al. 2017). Maximum-likelihood trees were constructed using IQ-Tree (1.6.12) with 1000 ultra-fast bootstrap replicates (Nguyen et al. 2015). For the *Nitrosomonas* tree, *Nitrosospira multiformis* ATCC 25196 (GCA_000196355.1) and *Nitrosospira briensis* C-128 (GCA_000619905.2) were used as outgroup, while *Leptospirillum ferriphilum* (GCA_900198525.1) and *Leptospirillum ferrooxidans* C2-3 (GCA_000284315.1) were used for the UBA8639 tree. For the *Nitrospinia* tree, the genomes of the following four *Nitrospira* species served as outgroup: *N. moscoviensis* NSP M-1 (GCF_001273775.1), *N. inopinata* ENR4 (GCF_001458695.1), *N. japonica* NJ11 (GCF_900169565.1), and *N. defluvii* (GCF_000196815.1). Finally, *Ca. Nitrososphaera gargensis* Ga9.2 (GCA_000303155.1), *Nitrososphaera viennensis* EN76 (GCA_000698785.1), *Ca*. Nitrosocosmicus oleophilus MY3 (GCA_000802205.2), *Ca*. Nitrosocosmicus arcticus Kfb (GCA_007826885.1), and *Ca*. Nitrosocosmicus franklandus C13 (GCA_900696045.1) were used for the outgroup of the *Nitrosopumilus* tree. Trees were visualized and annotated using the online interactive Tree of Life (iTol, v6) (Letunic and Bork 2016). Using the prodigal gene calling from the DRAM annotation pipeline (Hyatt et al. 2010, Shaffer et al. 2020), the average nucleotide (ANI) and average amino acid identities (AAI) of genomes with an estimated completeness of ≥90% and an estimated redundancy ≤5% were calculated using FastANI (Jain et al. 2018) and EzAAI (Kim et al. 2021) using default settings.

## Results and discussion

### 16S rRNA gene-based abundance and diversity of nitrifying populations

**Figure 1.**
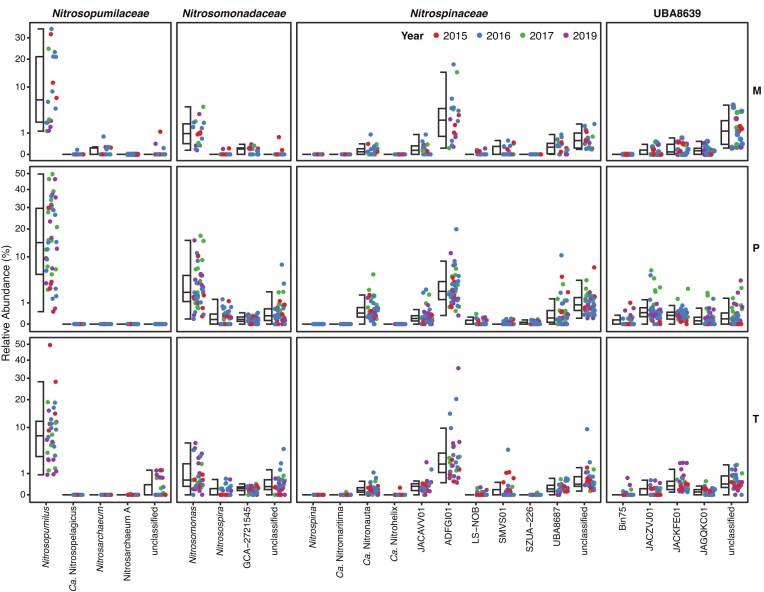
16S rRNA gene-based relative nitrifier abundances. Shown are all ASVs belonging to different genera within the known nitrifying families *Nitrosopumilaceae, Nitrosomonadaceae, Nitrospinaceae*, and UBA8639 (order *Nitrospirales*) in samples collected from biofilms of three different sites (P, pump station; T, test site; and M, main tunnel). The colors indicate the year the samples were collected. Note that the *y*-axis scales differ between rows.

The diversity of the microbial community growing in biofilms of the Oslofjord subsea tunnel was assessed by sequencing the 16S rRNA genes of bacteria and archaea. Samples (*n* = 95) were collected at three different locations (pump station (P), test site (T), and main tunnel (M)) in four different years (2015, 2016, 2017, and 2019). Putative nitrifiers were detected in all biofilm samples, although their relative abundance varied widely between samples with a minimum abundance of 1.2% and a maximum of 61.4%. The mean abundance of all putative nitrifier ASVs was 24.7 ± 15% (mean ± standard deviation). In total, we identified 53 ASVs affiliated with potential ammonia oxidizers (*Nitrosomonadaceae, Nitrosopumilaceae*), and 149 potential nitrite oxidizers (*Nitrospinaceae, Nitrospirales* family UBA8639). Among the nitrifying families, the relative abundance differed significantly (Friedman’s rank sum test, *P* < .001). The mean relative abundance of *Nitrosopumilaceae* was 14.1 ± 13%, ranging from 0.3% to 49.7% (Fig. 1). The relative abundance of *Nitrosomonadaceae* was significantly lower than that of *Nitrosopumilaceae* (Wilcoxon test, *P* < .001), with a mean abundance of 3.2 ± 4%, ranging from 0.09% to 18.1%. Furthermore, the relative abundance of *Nitrospinaceae* was significantly higher than that of the *Nitrospirales* family UBA8639 (*Nitrospira* sublineage IV; Wilcoxon test, *P* < .001). The mean relative abundance of *Nitrospinaceae* was 5.9 ± 6%, ranging from 0.2% to 38.1%, while the relative abundance of UBA8639 ranged from 0.02% to 8.1%, with a mean of 1.6 ± 2% (Fig. 1). Although the relatively high abundance of nitrifiers suggests that they play an important role in the tunnel biofilm microbial community, numerical abundance does not necessarily correlate with growth rates or activity (Kitzinger et al. 2020). Therefore, further studies are needed to quantify the contribution of each group to nitrification and their potential role in concrete biodeterioration in the biofilm.

Our analysis revealed substantial variation in ASV abundances between samples for all families, although samples were usually dominated by the same genera (Fig. 1). In the family *Nitrosopumilaceae*, ASVs belonging to the genus *Nitrosopumilus* were the most abundant at all locations (mean ± SD: 13.9 ± 13%; Fig. 1, Fig. S1). ASV1 of this genus was the most abundant in many samples at all locations, while ASV2 was more abundant in several samples from location P (Fig. S1). Within the family *Nitrosomonadaceae*, the majority of ASVs belonged to the genus *Nitrosomonas* (mean ± SD: 2.6 ± 3%), but no single *Nitrosomonadaceae* ASV was dominant. However, in several samples from location P, different ASVs of the genus *Nitrosomonas* prevailed (ASV56, ASV34, ASV19, and ASV17; Fig. S1). In the families *Nitrospinaceae* and UBA8639, the ASVs of several genera contributed to their abundance (Fig. 1). Within the *Nitrospinaceae*, the most abundant genus was JADFGI01 (3.8 ± 5%), with ASV7 being the most abundant ASV of this genus (Fig. S1). For the *Nitrospirales* family UBA8639, ASV25, which could not be classified to genus level, was the most abundant in several samples from location M, while ASV51 belonging to the genus JACZVJ01 (0.4 ± 1%) was more abundant in several samples from location P (Fig. S1).

### Correlations between microbial families

**Figure 2.**
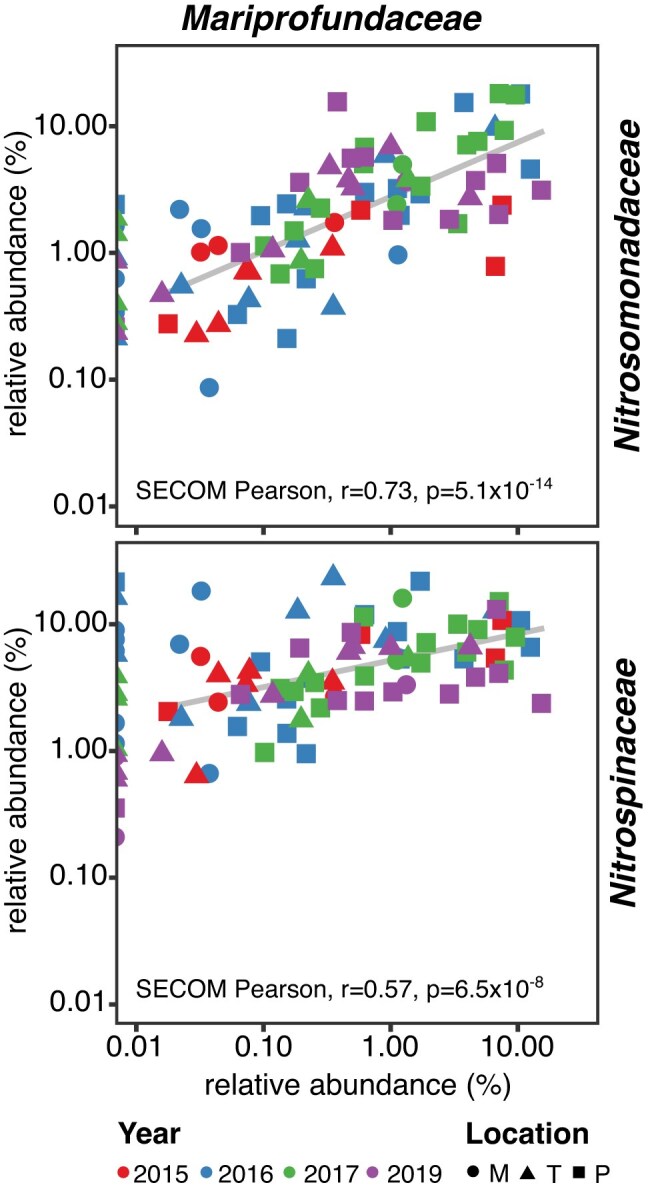
Linear correlations of the families *Nitrosomonadaceae* and *Nitrospinaceae* with the *Mariprofundaceae* based on 16S rRNA gene analysis. The colors and shapes of the symbols indicate the sampling year and location, respectively. Each shape shows the sum of the relative abundances of all ASVs belonging to the respective family.

To gain further insight into community dynamics and potential interactions, we grouped all individual ASVs on the family level and used their abundances across the different samples to assess linear relationships between nitrifiers and other taxa (Fig. 2, Figs S2 and S3, and Table S2). The relative abundance of the ammonia-oxidizing *Nitrosomonadaceae* was positively correlated with both nitrite-oxidizing families present in the biofilm, the *Nitrospinaceae* and the *Nitrospirales* family UBA8639. In contrast, the ammonia-oxidizing archaea (AOA; family *Nitrosopumilaceae*) were only weakly correlated with these nitrite oxidizers. The strongest correlation between nitrifying and non-nitrifying families was observed between the *Nitrosomonadaceae* and *Mariprofundaceae* (SECOM Pearson, *r* = 0.73), and also the *Nitrospinaceae* ASVs were correlated with this family (SECOM Pearson, *r* = 0.57; Fig. 2). Furthermore, positive correlations were observed between *Nitrosomonadaceae* and *Rhodobacteraceae*, between the *Nitrospinaceae* and *Vampirovibrionaceae*, as well as the *Nitrosopumilaceae* and *Phycisphaeraceae*. Conversely, strong negative correlations were found between the *Nitrosomonadaceae* and the families UBA2386 (*Planctomycetota*), UBA2774 (*Desulfobacterota*), and GWE2-31-10 (*Spirochaetota*; Fig. S3, Table S2).

### Metagenomic analysis of nitrifier diversity

#### Genome-centric analysis confirms high nitrifier diversity

**Figure 3.**
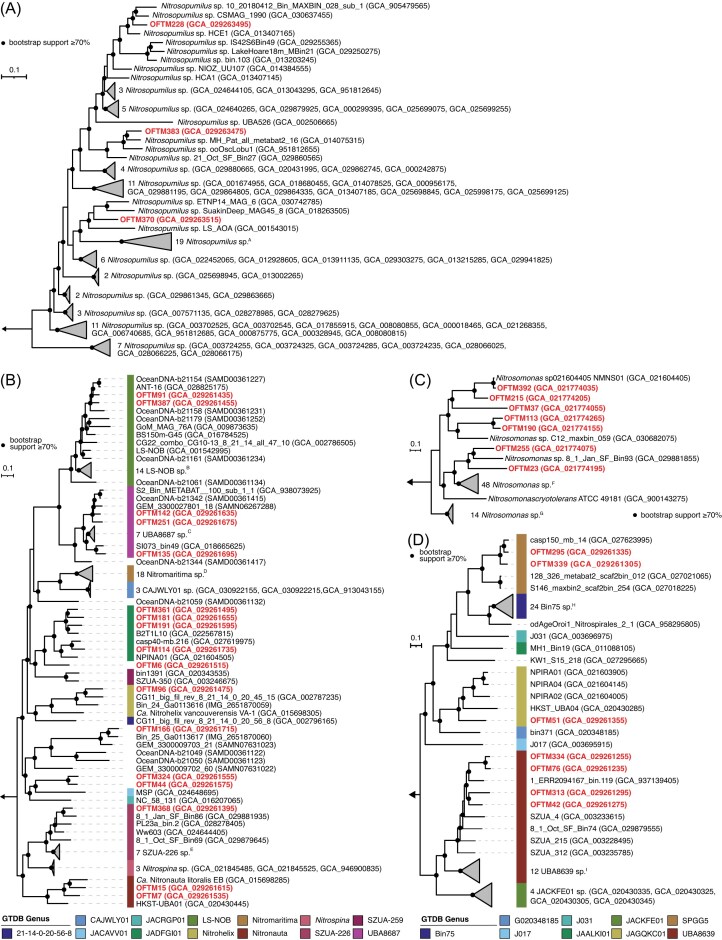
Phylogenetic affiliation of the nitrifying MAGs. Phylogenomic maximum-likelihood trees of the genus *Nitrosopumilus* (A), class *Nitrospinia* (B), genus *Nitrosomonas* (C), and the *Nitrospirales* family UBA8639 (D). The bacterial trees (B–D) are based on concatenated alignments of 92 core protein sequences, whereas the *Nitrosopumilus* tree (A) is based on a concatenated alignment of 36 ribosomal protein sequences. Black circles represent bootstrap support ≥70% of 1000 ultrafast bootstrap replicates. For the class *Nitrospinia* (B) and the family UBA8639 (D), the genus classifications according to GTDB-Tk with the r214 database are shown by colored bars. MAGs recovered from the tunnel biofilm are marked in bold red. Some sequence accessions have been omitted, see full lists of accession numbers of the collapsed tree branches in Table S7.

Similar to the 16S rRNA gene analyses, genome-centric metagenomic analyses also revealed a remarkable diversity of nitrifiers in the biofilm samples (Fig. 3). In total, we identified 10 MAGs of potential ammonia oxidizers and 24 potential nitrite oxidizers. In general, the ASV data and the recovered MAGs matched well but some discrepancies in the recovered genera were observed (Fig. S4). For example, the 10 ammonia oxidizer MAGs (≥50% completeness, ≤10% redundancy) represented seven different species of the genus *Nitrosomonas* and three *Nitrosopumilus* species (Fig. 3A and 2C, Figs S5 and S6). 16S rRNA gene sequencing, in contrast, detected ASVs from at least five and four different genera of *Nitrosopumilaceae* (*Nitrosopumilus, Nitrosopelagicus, Nitrosarchaeum*, Nitrosarchaeum_A, and several ASVs unclassified at the genus level) and *Nitrosomonadacae* (*Nitrosomonas, Nitrosospira*, GCA-2721545, and several ASVs unclassified at the genus level) in the samples, respectively. Similarly, within the class *Nitrospinia*, we identified MAGs of 17 novel species belonging to 8 different genera (LS-NOB, UBA8687, *Nitrohelix*, JAGFGI01, SZUA-226, *Nitronauta*, and two unnamed genera tentatively called Bin_25 and 702_60; Fig. 3B, Fig. S7), but additional ASVs were found belonging to the genera *Nitrospina, Ca*. Nitromaritima, JACAVV01, and SMVS01. Within the *Nitrospirales* family UBA8639, seven MAGs were recovered that included four MAGs from the genus UBA8639 and one belonging to the genus JAGQKC01, but also two MAGs of the genus SPGG5 not represented by ASVs (Fig. 3D, Fig. S8). In contrast, the ASVs within this family affiliated with four genera (Bin75, JACKFE01, JACZVJ01, and JAGQKC01) or remained unclassified on genus level, with the genera Bin75, JACKFE01, or JACZVJ01 not represented in the recovered MAGs.

#### Metabolic potential of nitrifying populations

**Figure 4.**
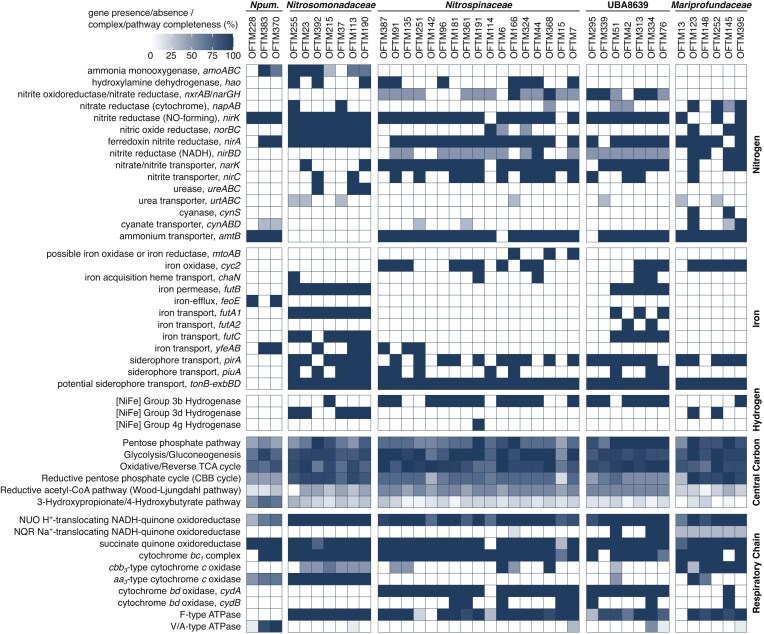
Functional MAG annotation. The heatmap indicates the presence and completeness of genes and pathways involved in nitrogen, iron, and hydrogen metabolism, as well as the central carbon metabolism and CO_2_-fixation pathways and the respiratory chain. Annotations are shown for MAGs belonging to the families *Nitrosopumilaceae* (*Npum*.) *Nitrosomonadaceae, Nitrospinaceae*, UBA8639 (order *Nitrospirales*), and *Mariprofundaceae*.

Not all nitrifier MAGs recovered contained the key functional genes *amoABC, hao*, or *nxrABC* (Fig. 4). However, their phylogenetic affiliation with known nitrifying families (Fig. 3, Figs S5–S8) suggested that the absence of these genes is likely due to genome incompleteness. In addition, we found that the core metabolisms of all nitrifier MAGs (Fig. 4) are highly similar to those of previously described members of these families (Chain et al. 2003, Arp et al. 2007, Lücker et al. 2010, 2013, Walker et al. 2010, Palomo et al. 2018, Wright and Lehtovirta-Morley 2023) and will therefore not be discussed here.

#### Hydrogen metabolism might be widespread

Several *Nitrosomonadacae, Nitrospinaceae*, and *Nitrospirales* family UBA8639 MAGs were found to possess putatively O_2_-tolerant group 3b and 3d [NiFe] hydrogenases (Fig. 4). Hydrogen oxidation could serve as an alternative energy conservation strategy for these organisms, allowing them to utilize H_2_ produced during steel corrosion (Mori et al. 2017) or by fermentation in anoxic layers of the biofilm. Hydrogen oxidation has been documented in ammonia oxidizers of the genus *Nitrosomonas* (Bock et al. 1995) and in the nitrite oxidizer *Nitrospira moscoviensis*, which uses a group 2a [NiFe] hydrogenase for hydrogenotrophic growth and oxidation of hydrogen even under atmospheric levels (Koch et al. 2014, Leung et al. 2022). Hydrogen oxidation may provide nitrifiers with an additional energy source when nitrite concentrations fluctuate, and even in the presence of nitrite can serve as a source of electrons for CO_2_ fixation (Koch et al. 2014, Leung et al. 2022). Still, evidence for hydrogen oxidation using the group 3 [NiFe] hydrogenases in nitrifiers is lacking.

#### Potential for metal oxidation

Several MAGs belonging to the nitrite-oxidizing families *Nitrospinaceae* and UBA8639 contained *cyc2*-like genes encoding potential iron oxidases, and three additional *Nitrospinaceae* MAGs (OFTM166, OFTM368, and OFTM7) encoded the potential iron oxidase MtoAB (Fig. 4) (Liu et al. 2012). Furthermore, putative manganese oxidases (MoxA, MnxG, and McoA) were found in almost all biofilm nitrifier MAGs (Fig. S9). These genes were also found in published genomes of the *Nitrosomonadaceae, Nitrosopumilaceae, Nitrospinaceae*, and UBA8639 families (data not shown), including those of cultured species. However, manganese oxidation capacity has not yet been demonstrated for any member of these families, and, considering the substrate promiscuity of many MCOs, their function in these nitrifiers awaits experimental verification. Besides energy conservation, manganese cycling by MCOs can also play a role in the protection from reactive oxygen species, which can be neutralized by manganese oxides (Daly 2009).

Metal-reducing or oxidizing microorganisms often employ multiheme cytochrome *c* proteins that transfer electrons to or from metal(hydr)oxides, which are not able to permeate through the cell envelope (Shi et al. 2016). Although with no similarity to the proteins of known metal reducers or oxidizers (Simon and Klotz 2013), many nitrifier MAGs encode multiheme cytochrome *c* proteins, and gene clusters with several (≥3) multiheme cytochrome *c* genes with up to 12 heme-binding sites were encoded in several *Nitrospinacaeae* MAGs (OFTM368, OFTM387, OFTM6, and OFTM96; Table S1). While these proteins may function in extracellular electron transport and allow interaction with iron or manganese particles, the role of these gene clusters remains unclear due to the lack of physiological data.

#### Motility and virus defense

Almost all MAGs of the *Nitrosomonadaceae* and nitrite oxidizing families UBA8639 and *Nitrospinaeace* encoded genes for flagellum biosynthesis and assembly (Table S1). In addition, we identified complete chemotaxis pathways in UBA8639 and *Nitrosomonadaceae* MAGs. The presence of aerotaxis receptors indicated the ability of these organisms to exhibit aerotactic behavior by moving along oxygen gradients. These findings highlighted the potential of these nitrifying organisms to sense and respond to their environment within the biofilm matrix.

Interestingly, although the biofilm environment is expected to promote increased microbe–virus interactions (Zhang et al. 2019), our analysis revealed only a limited presence of CRISPR arrays and Cas proteins in the nitrifier MAGs (Table S3).

### Diversity and metabolic potential of *Mariprofundaceae*

Due to the strong correlations of the *Nitrosomonadaceae* and *Nitrospinaceae* with the *Mariprofundaceae*, we analyzed their diversity and genomic potential in more detail. In the 16S rRNA gene-based analysis, we recovered 48 ASVs within the *Mariprofundaceae* belonging to at least four different genera (WSZY01, *n* = 23; *Mariprofundus, n* = 17; GCA-2401635, *n* = 2; unclassified, *n* = 6), while metagenomics returned 6 MAGs belonging to the genera *Ghiorsea* (*n* = 1), *Mariprofundus* (*n* = 4), and WSZY01 (*n* = 1; Fig. S4). This family belongs to the class *Zetaproteobacteria* and all available isolates are obligate autotrophs capable of microoxic Fe(II) oxidation (McAllister et al. 2019). Previous studies have identified *Zetaproteobacteria* in corrosion biofilms, where they are usually early colonizers (McBeth et al. 2011, Lee et al. 2013, McBeth and Emerson 2016, Mumford et al. 2016). Fe^2+^ oxidation leads to the production of Fe^3+^ oxyhydroxide stalks, forming a porous matrix similar to observations in the Oslofjord tunnel samples (Hagelia 2011, Karačić et al. 2018). Accordingly, all high-quality *Mariprofundaceae* genomes encoded putative Fe(II) oxidases, high-affinity terminal cytochrome *c* oxidases, and the Calvin–Benson–Bassham cycle for CO_2_ fixation for autotrophic growth, and diverse defense systems against reactive oxygen species (ROS; see Supplementary Text for more details), which is a common feature of corrosion-associated *Zetaproteobacteria* (Field et al. 2015). Furthermore, like the nitrifiers, several *Mariprofundaceae* MAGs were found to possess group 3b and 3d [NiFe] hydrogenases (Fig. 4). Some *Mariprofundaceae* belonging to the genus *Ghiorsea* are capable of simultaneous hydrogen and Fe^2+^ oxidation (Mori et al. 2017), indicating that also in this biofilm system hydrogen might serve as an alternative energy source.

Corrosion of the concrete steel reinforcements may not only be directly promoted by iron oxidation by *Mariprofundaceae*, but their growth in biofilms may play an additional role in enhancing corrosion. One of the *Mariprofundus* MAGs (OFTM13) contained a complete *wsp* gene cluster (*wspABCDEFR*) for the Wsp chemosensory system (Table S4), which is involved in biofilm formation and has been previously observed in two *Mariprofundus* species (Chiu et al. 2017). EPS commonly produced by biofilm-forming bacteria can bind metal ions such as Ca^2+^, Cu^2+^, Mg^2+^, and Fe^3+^. Electrons from the zero-valent Fe can be directly transferred to EPS-bound Fe^3+^, oxidizing the zero-valent Fe and reducing the Fe^3+^ to Fe^2+^. The formed Fe^2+^ can then, in the presence of O_2_, be abiotically reoxidized to Fe^3+^, thus resulting in enhanced corrosion (Beech and Sunner 2004).

#### Nitrogen metabolism could connect Mariprofundaceae and nitrifiers

We found diverse nitrogen use and acquisition strategies within the biofilm *Mariprofundaceae*. Several of these MAGs contained genes encoding nitrate reductase (NapAB), NO-forming nitrite reductase (NirK), and nitric oxide reductase (NorBC), allowing potential denitrification of nitrate to nitrous oxide (Fig. 4). In addition, diverse nitrogen acquisition pathways were present in the MAGs. Besides nitrate reductase, we found genes for nitrate/nitrite transport (*narK*), assimilatory nitrite reduction to ammonium (*nirA, nirBD*), and ammonium uptake (*amtB*). Two of the MAGs (OFTM123, genus *Mariprofundus*; OFTM145, genus *Ghiorsea*) also had the potential for cyanate breakdown (*cynS*), giving them the potential to switch between organic and inorganic nitrogen sources (Fig. 4). The potential of *Mariprofundaceae* to use nitrate or nitrite as electron acceptor and nitrogen source may explain the positive correlation with the nitrifying families *Nitrosomonadaceae* and *Nitrospinaceae*. However, the exact nature of this interaction, as well as its potential benefits for especially the nitrifying partner, remains to be experimentally tested.

#### Amino acid and vitamin B_12_ biosynthesis

Interestingly, the majority of *Mariprofundaceae, Nitrosomonadaceae*, UBA8639, and *Nitrospinaceae* MAGs lacked a complete cobalamin (vitamin B_12_) biosynthetic pathway. Instead, they only encoded the pathway to convert the precursor cobyrinate to cobalamin (Fig. S10). This has previously also been observed in *Nitrospira marina* and *Nitrospinaceae* (Bayer et al. 2021, Mueller et al. 2021). Thus, it appears that these bacteria rely on obtaining cobalamin or its precursors from the environment. Interestingly, three *Nitrospinaceae* MAGs contained the almost complete anaerobic cobalamin biosynthesis pathway, with only one or two genes missing, probably due to genome incompleteness, and one even encoded the complete pathway (Fig. S10). In addition, two of the three *Nitrosopumilaceae* MAGs also had a nearly complete cobalamin biosynthesis pathway. These few cobalamin producers may fulfill a Black Queen function within the biofilm communities, where increased fitness by genome streamlining is enabled by “helper” strains that produce common goods (Morris et al. 2012, Mas et al. 2016), which may also contribute to the observed high nitrifier diversity.

None of the nitrifier or *Mariprofundaceae* MAGs encoded complete tyrosine and phenylalanine biosynthetic pathways (Fig. S10), suggesting that they use unknown biosynthetic pathways or rely on the production of these amino acids by other members of the community, which may explain their positive correlations (Table S3). The *Nitrosopumilaceae* MAGs lacked or had incomplete biosynthetic pathways for several amino acids (glycine, tyrosine, phenylalanine, proline, methionine, and cysteine). However, a comparison with genomes of cultured representatives capable of chemolithoautotrophic growth (not shown) suggests this was likely due to incomplete gene annotation rather than an actual lack of biosynthetic pathways, as it is likely that the genes for archaeal amino acid biosynthesis are not well annotated, or that they use alternative pathways.

#### Protection against toxic metals and metalloids

Seawater seeping over the biofilms is relatively rich in metals and metalloids (Karačić et al. 2018), and nitrifiers, as well as MAGs of correlating families, appeared to be well-equipped to deal with these potentially toxic compounds. Genes encoding heavy metal efflux systems were found in the majority of the analyzed genomes, along with zinc transporters, mercuric reductases and transporters, and arsenate reductases (Fig. S9).

### Elucidating the interactions between nitrifiers and *Mariprofundaceae*

In summary, positive correlations between ASVs of nitrifying families and the *Mariprofundaceae* might be explained by several factors. For example, nitrifiers may provide nitrogen compounds and vitamin B_12_ to their interaction partners, fulfilling a Black Queen function within the community. Conversely, *Mariprofundaceae* may be early biofilm formers providing nitrifiers with a protected environment, and nitrifiers might also benefit from the Fe^3+^-mediated oxidation of ferrous metal by the EPS forming the biofilms, providing them with Fe^2+^. Other microbes in the biofilms may provide amino acids. However, metagenomic analyses alone will rarely inform about the nature of the observed correlations between nitrifiers and other microbial groups. Here, further studies are needed to, for instance, investigate their spatial organization within the biofilms, as spatial patterns can better reveal cooperative and competitive interactions between microbes (Daims et al. 2006, Nadell et al. 2016). Combining fluorescence *in situ* hybridization (FISH) with microsensors, or with methods visualizing their function like, for instance, microautoradiography or Raman microspectroscopy, can elucidate the distribution of microbial groups and provide further insights into their metabolism (Ramsing et al. 1993, Schramm et al. 1996, Okabe et al. 1999, 2004, Daims et al. 2001, Lee et al. 2021). Omics techniques can be applied to study interspecies relationships (Ellepola et al. 2019), but are complicated by the complexity of biofilm communities. However, a pipeline combining mass spectrometry imaging and FISH has been developed to link metabolites to community members (Geier et al. 2020). It could be used to study interspecies interactions and metabolic phenotypes of populations at the resolution of small cell clusters, but this technique has yet to be applied and tested on biofilm samples.

### Ecological implications

The observed high diversity of and within functional groups appears paradoxical, as these species are expected to compete for the same limited substrates. Previous studies have observed dominance of either AOA or ammonia-oxidizing bacteria (AOB) and the abundance of a single type of nitrite oxidizer in the pelagic open ocean (Mincer et al. 2007, Levipan et al. 2014, Lau et al. 2019) or thermal spring biofilm samples (Marks et al. 2012), while for marine sediments the coexistence of phylogenetically diverse but functionally redundant nitrifier groups was reported (Reyes et al. 2017, Hollingsworth et al. 2021). This coexistence of various nitrifier groups might suggest that they have distinct properties that allow niche differentiation. Similarly, differential abundances of sulfur-oxidizing bacteria and methane oxidizers in other ecosystems were previously found to be explained by substrate concentrations and temperature (Meier et al. 2017, Mayr et al. 2020). Moreover, fine-scale spatial segregation within the biofilm matrices could lead to the existence of distinct niches for organisms within the same functional group (Maixner et al. 2006, Louca et al. 2018), as corroborated here by the high variability between samples from the same locations (Fig. 1, Fig. S1). Here, high resolution sampling of the biofilms in combination with physicochemical measurements could help identify such drivers of the niche differentiation among nitrifiers. The limited occurrence of ubiquitous ASVs (present in ≥80% of the samples) and the predominance of occasional nitrifier ASVs (present in <20% of the samples; Table S6) further supports that specialization also within nitrifier families results in the occupation of different niches. Several factors may contribute to niche differentiation, including affinity for ammonia or nitrite, oxygen tolerance and affinity, use of different nitrogen sources for assimilation, nutritional auxotrophies, and pH range (Hatzenpichler 2012, Qin et al. 2014, Hou et al. 2018). In particular, while nitrifiers will compete with heterotrophs for ammonium, nitrite, and/or oxygen, nitrifying subpopulations also can have distinct preferences for prevailing ammonium and nitrite concentrations, contributing to niche differentiation within the biofilms (Stein and Arp 1998, Maixner et al. 2006, Almstrand et al. 2013, Gruber-Dorninger et al. 2015, Ushiki et al. 2017, Reji et al. 2019). In addition, the ability to utilize organic substrates and hydrogen may also be a niche-defining property (Daims et al. 2001, Koch et al. 2014, Qin et al. 2014, Gruber-Dorninger et al. 2015). Furthermore, the availability of iron and copper may play a role in structuring the community of ammonia oxidizers. While AOB use a greater number of iron-based enzymes, AOA encode numerous copper-containing enzymes and appear to have a reduced affinity for iron (Amin et al. 2013, Shafiee et al. 2019), and some nitrifiers may rely on siderophore production by other organisms for iron acquisition (Chain et al. 2003, Keluskar et al. 2013). Other factors such as chemotactic properties, adaptations to environmental stresses, predation, variation in antibiotic resistance, and susceptibility to phages could also influence community composition (Rodriguez-Valera et al. 2009, Dolinšek et al. 2013, Louca et al. 2018, Sampara et al. 2022).

Microbial diversity and intrahabitat variation are often excessive in biofilms (Zhang et al. 2019), as has also been observed in biofilm communities growing on concrete (Karačić et al. 2022). The high diversity may increase resilience to fluctuating environmental conditions, known as the “insurance hypothesis” (Yachi and Loreau 1999). Microcosm experiments have shown that genotypic and functional diversity within a community increases stability in the face of biotic or abiotic perturbations (Eisenhauer et al. 2012). Consistent with this, long-term monitoring at a wastewater treatment plant found significant temporal variation in the abundance of nitrifying species, while nitrification rates remained stable (Wells et al. 2009). Even during short-term growth in biofilms extensive genetic diversification can take place, resulting in variations in motility, nutritional requirements, production of secreted products, and biofilm phenotypes, ultimately leading to improved survival and resistance to environmental stresses (Boles et al. 2004). Similarly, the high diversity between and within the nitrifying biofilm communities investigated here may result in increased resilience to ecosystem disturbances.

## Conclusions

Here, we show that nitrifiers are abundant and highly diverse in the biofilms growing on the steel-reinforced concrete walls of the subsea tunnel under the Oslofjord. We suggest that this coexistence of surprisingly many different groups of nitrifiers could be explained by their variable metabolic potential and the existence of fine-scale environmental niches within the biofilms, which warrants high-resolution sampling and physicochemical measurements. In addition, their role in concrete degradation, as well as our hypothesis that the high nitrifier diversity could lead to increased resilience of the microbial community, could be tested using mesocosm experiments in the future. We identified a strong positive correlation between nitrifying families and several *Mariprofundaceae* species and hypothesized that nitrifiers could provide nitrogen compounds and vitamin B_12_ to associated bacteria in exchange for a protected environment. The insights from the ASV and MAG-based analyses open new avenues for further research on microbial communities, which is ultimately important for understanding the role of such biofilms in concrete biodeterioration.

## Supplementary Material

fiaf032_Supplemental_Files

## Data Availability

The 16S rRNA gene sequence reads are available at NCBI under the Bioproject accession numbers PRJNA481470 and PRJNA1061464. Raw metagenome reads and MAGs are available under the NCBI Bioproject accession number PRJNA755678. All other relevant data has been made available as Zenodo repository (https://zenodo.org/records/14039362).
