# Correction to: High diversity of nitrifying bacteria and archaea in biofilms from a subsea tunnel

**DOI:** 10.1093/femsec/fiaf092

**Published:** 2025-09-22

## Abstract

Shredder-derived fine particulate organic matter hosts distinct bacterial communities influenced by conditioning and gut passage, highlighting its role in enhancing microbial diversity in stream ecosystems.

This is a correction to: Linnea F M Kop, Hanna Koch, Paula Dalcin Martins, Carolina Suarez, Sabina Karačić, Frank Persson, Britt-Marie Wilén, Per Hagelia, Mike S M Jetten, Sebastian Lücker, High diversity of nitrifying bacteria and archaea in biofilms from a subsea tunnel, *FEMS Microbiology Ecology*, Volume 101, Issue 5, May 2025, fiaf032, https://doi.org/10.1093/femsec/fiaf032.

In the originally published version of this manuscript, Figure 2 was omitted and Figure S2 was erroneously included in the main text in its place.

The manuscript has now been updated to include the correct version of Figure 2.

