# Supplementary material for: High diversity of nitrifying bacteria and archaea in biofilms from a subsea tunnel
Source: FEMS Microbiol Ecol. 2025 Mar 28;101(5):fiaf032. doi: 10.1093/femsec/fiaf032 (PMC11995701; doi:10.1093/femsec/fiaf032)
Supplement: fiaf032_Supplemental_Files [file fiaf032_supplemental_files.zip › Kop2025_FEMS_ME_Supplementary_Materials.pdf]

High diversity of nitrifying bacteria and archaea in biofilms from a subsea tunnel

Linnea F.M. Kop[1], Hanna Koch[1,2], Paula Dalcin Martins[3], Carolina Suarez[4], Sabina Karačić[5,6], Frank Persson[5], Britt-Marie Wilén[5], Per Hagelia[7,8], Mike S.M. Jetten[1], Sebastian Lücker[1,*]

[1]Department of Microbiology, Radboud Institute for Biological and Environmental Sciences, Radboud University, Heyendaalseweg 135, 6525 AJ, Nijmegen, the Netherlands

[2]Bioresources Unit, Center for Health & Bioresources, AIT Austrian Institute of Technology GmbH, Konrad-Lorenz-Straße 24, 3430 Tulln an der Donau, Austria

[3]Ecosystems & Landscape Dynamics, Institute for Biodiversity and Ecosystem Dynamics, University of Amsterdam, Science Park 904, 1090 GE Amsterdam

[4]Division of Water Resources Engineering, Faculty of Engineering LTH, Lund University, John Ericssons väg 1, 221 00 Lund, Sweden

[5]Division of Water Environment Technology, Department of Architecture and Civil Engineering, Chalmers University of Technology, Sven Hultins gata 6, 412 96 Gothenburg, Sweden

[6]Institute of Medical Microbiology, Immunology and Parasitology, Universitätsklinikum Bonn, Venusberg – Campus 1, 53127 Bonn, Germany

[7]Construction Division, The Norwegian Public Roads Administration, Innspurten 11C, 0663 Oslo, Norway

[8]Müller-Sars Biological Station, Ørje, P.O. Box 64, NO-1871 Ørje, Norway

* Corresponding author: Sebastian Lücker, Department of Microbiology, Radboud Institute for Biological and Environmental Sciences, Radboud University, Heyendaalseweg 135, 6525 AJ Nijmegen, The Netherlands. Email: s.luecker@science.ru.nl

Supplementary Materials

This document contains Supplementary Results and Discussion and Supplementary Figures S1-S10. Supplementary Tables S1-S7 are provided in a separate Excel file.

Supplementary Results and Discussion

Metabolic potential of Mariprofundaceae

Putative Fe(II) oxidases with predicted secretory signal peptides and CXXCH heme-binding motifs were identified in all *Mariprofundaceae* genomes except for the medium-quality MAG OFTM13 (genus *Mariprofundus*; Figure 4, Figure S9). In addition, they encoded the Calvin-Benson-Bassham cycle for CO_2 fixation to allow autotrophic growth. Most MAGs contained a high-affinity *cbb*$_3$-type cytochrome *c* oxidase for aerobic respiration of Fe(II) or H_2. The MAG OFTM145 (genus *Ghiorsea*) additionally encoded a cytochrome *bd* oxidase (CydAB), which has also been described to have a high O_2 affinity but accepts electrons from the quinol pool (Borisov *et al.* 2011). Similar to other iron oxidizers (Emerson *et al.* 2013), some of the *Mariprofundaceae* MAGs encoded hemerythrin and hemoglobins that bind O_2, which may allow them to persist under low O_2 conditions in the deeper layers of the biofilm where they can access Fe^{2+} released from the tunnel steel reinforcement.

Another feature of corrosion-associated *Zetaproteobacteria* is the presence of defense systems against reactive oxygen species (ROS), which are generally rare in iron-oxidizing bacteria (Field *et al.* 2015). Correspondingly, all of the recovered *Mariprofundaceae* MAGs encoded a cytochrome *c* peroxidase, and the majority of them also contained glutaredoxins or peroxiredoxin for ROS defense. In addition, OFTM123 encoded superoxide dismutase, catalase, and thioredoxin (Supplementary Table S4). Additionally, the abovementioned hemerythrin and hemoglobins may prevent ROS formation by binding excess O_2 (Frey and Kallio 2003).

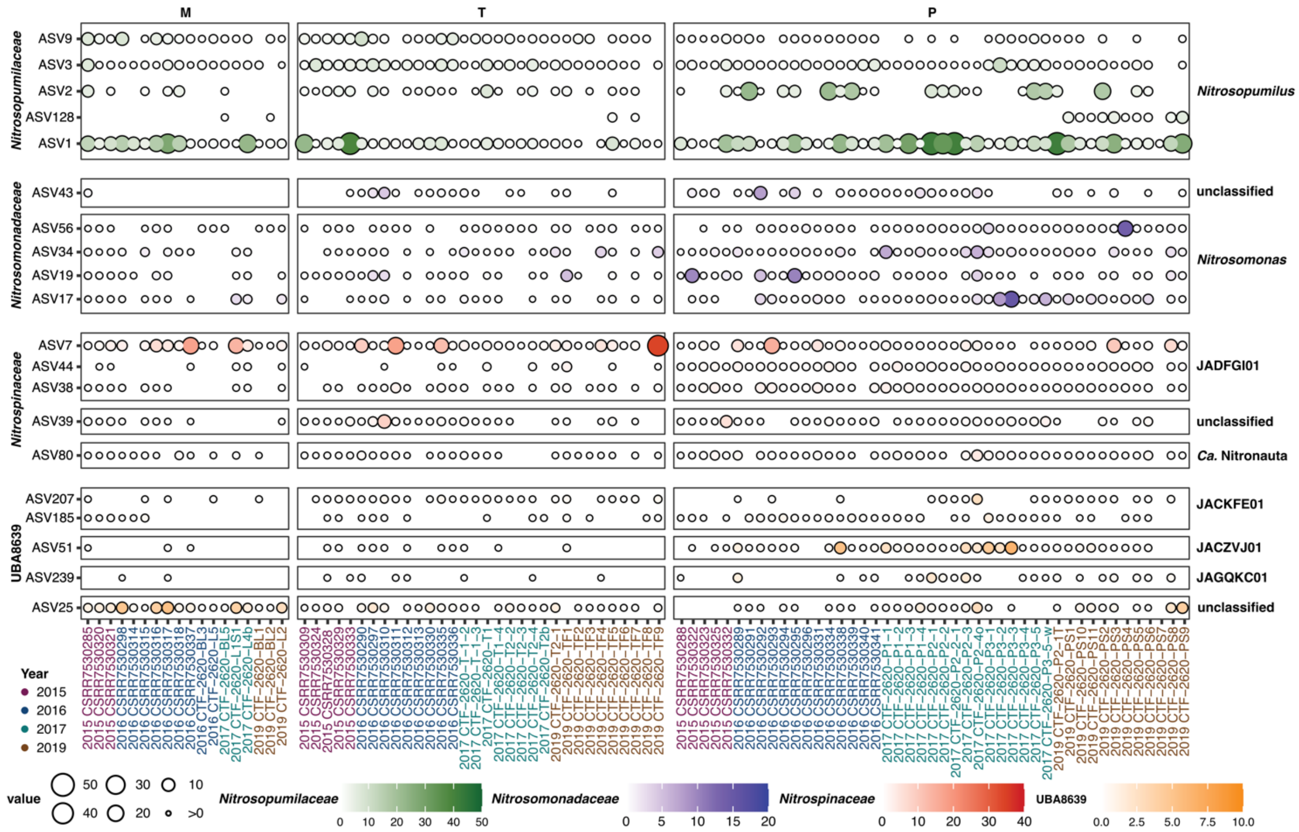

Figure S1. Relative abundances of the most abundant ASVs belonging to different genera within the known nitrifying families *Nitrosomonadaceae*, *Nitrosopumilaceae*, *Nitrospinaceae*, and UBA8639 (order *Nitrospirales*) in different samples. The labels on the left show the family, those on the right the genus classification for each ASV. Sample names are colored according to the year of sampling. The relative abundances of all ASVs were summed across all samples and the five most abundant ASVs per family are shown here.

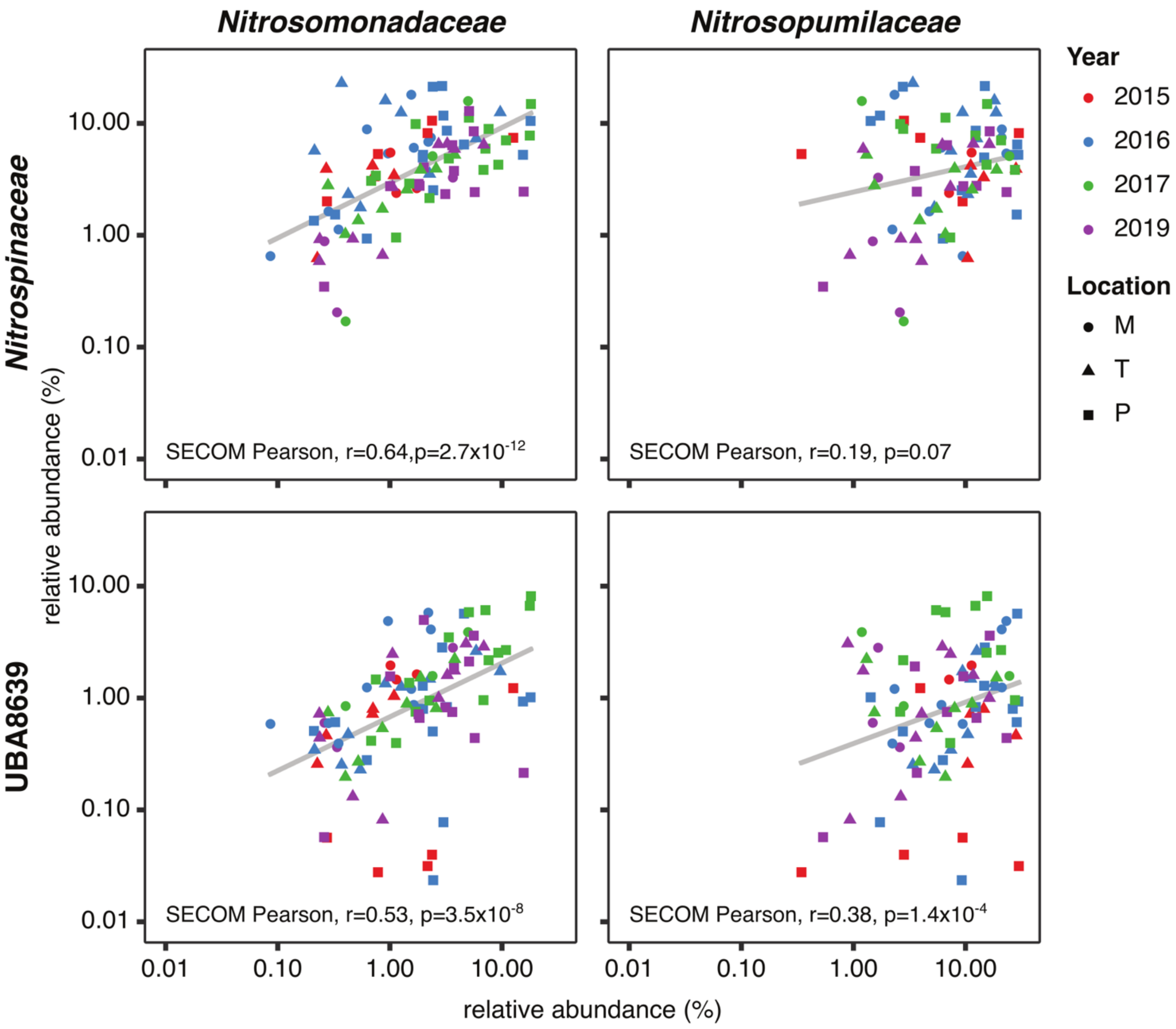

Figure S2. Linear Pearson correlations between relative abundances of the four nitrifying families *Nitrosomonadaceae*, *Nitrosopumilaceae*, *Nitrospinaceae*, and UBA8639 (order *Nitrospirales*) based on 16S rRNA gene analysis. The colors and shapes of the symbols indicate sampling year and location, respectively. Each shape shows the sum of the relative abundances of all ASVs belonging to the respective family.

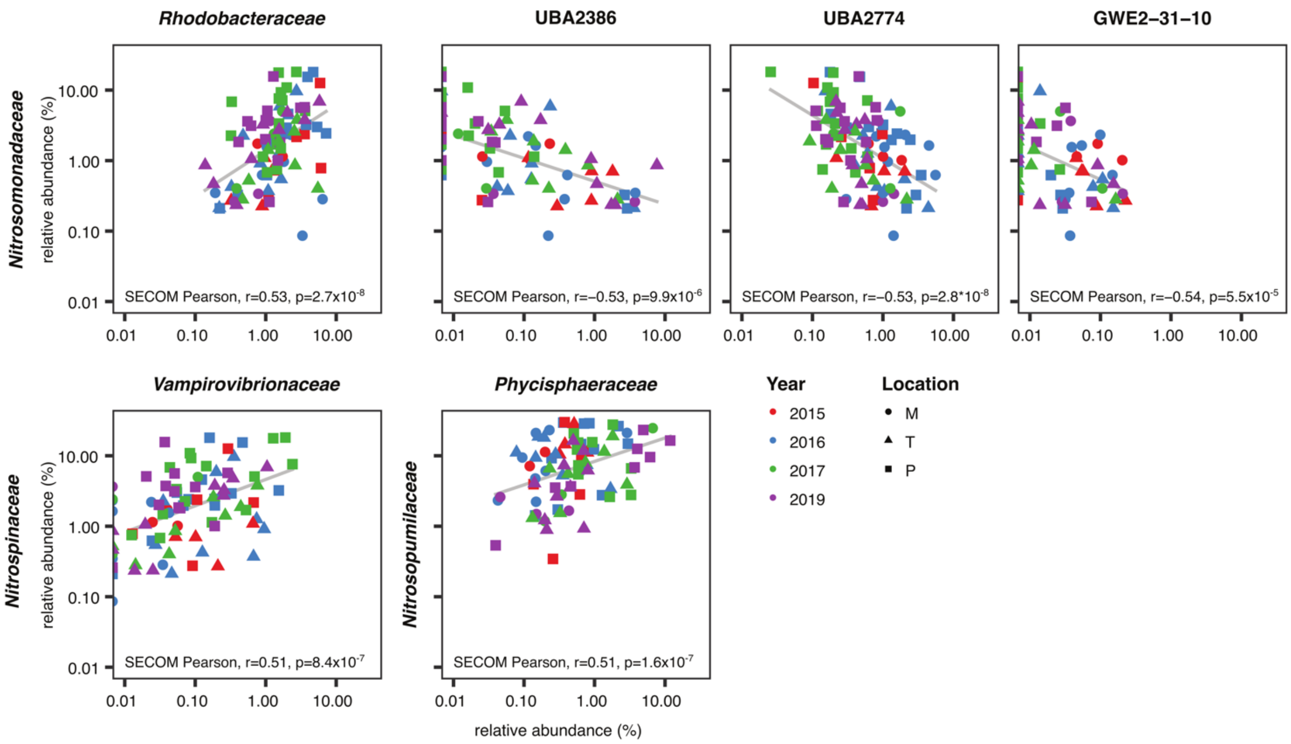

Figure S3. Correlations between relative abundances of the nitrifying families *Nitrosomonadaceae*, *Nitrospinaceae*, and *Nitrosopumilaceae*. Linear Pearson correlations were calculated using SECOM with the ANCOMBC R package using the secom_linear function with thresholding (Lin et al., 2022; Lin and Peddada, 2020). Correlations ≥0.5 and ≤-0.5 are shown, for further correlation values, see Supplementary Table S2. The colors and shapes of the symbols indicate sampling year and location, respectively. Each shape shows the sum of the relative abundances of all ASVs belonging to the respective family. The families UBA2386, UBA2774, and GWE2-31-10 belong to the phyla *Planctomycetota*, Desulfobacterota_D, and *Spirochaetota*, respectively.

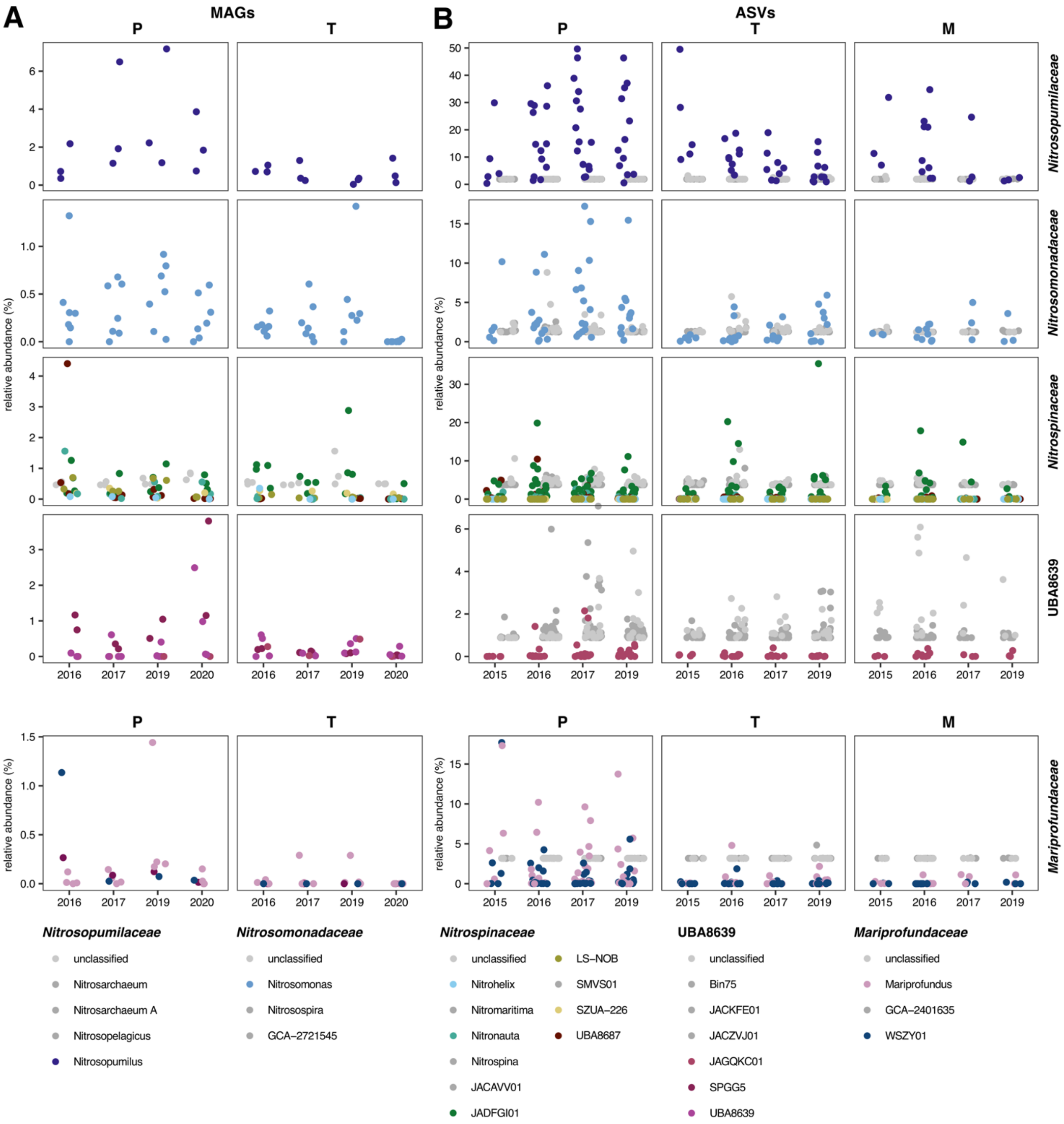

Figure S4. Relative abundance of MAGs (A) and ASVs (B) belonging to the families *Nitrosomonadaceae*, *Nitrosopumilaceae*, *Nitrospinaceae*, UBA8639 (order *Nitrospirales*), and *Mariprofundaceae*. Unclassified genera are shown in light gray. Genera depicted in dark gray are represented by ASVs only.

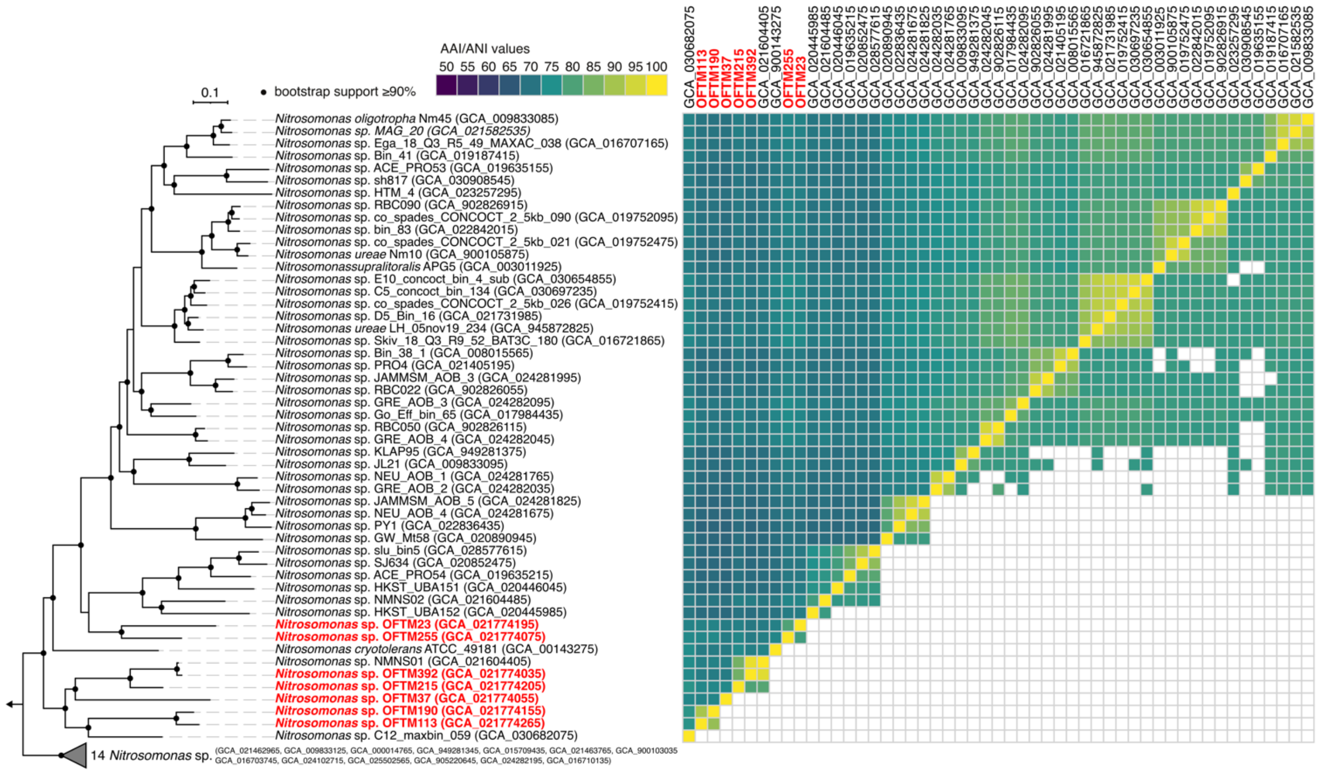

Figure S5. On the left, a phylogenomic tree of dereplicated high quality (≥90% completeness, ≤5% redundancy) genus *Nitrosomonas* genomes based on concatenated alignments of 92 core protein sequences is shown. The maximum likelihood tree was calculated using IQ-tree with the GTR+F+I+G4 model selected by ModelFinder. Black circles represent bootstrap support ≥70% of 1000 ultrafast bootstrap replicates. MAGs obtained in this study are shown in red. On the right, average nucleotide identities (ANI) and average amino acid identities (AAI) are shown. The lower right part of the heatmap shows AAI values, the upper left part shows ANI values ≥70%; ANI values <70% are replaced by white squares.

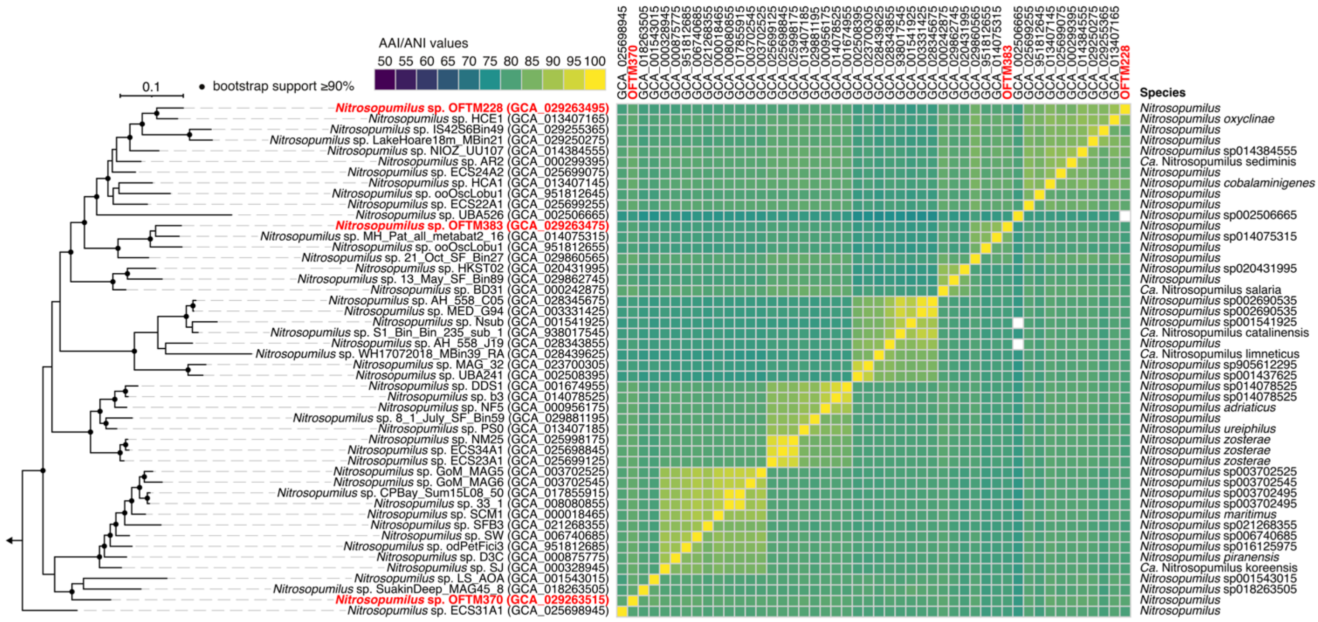

Figure S6. On the left, a phylogenomic tree of dereplicated high quality (≥90% completeness, ≤5% redundancy) genus *Nitrosopumilus* genomes based on concatenated alignments of 36 archaeal ribosomal genes is shown. The maximum likelihood tree was calculated using IQ-tree with the JTTDCMut+F+I+G4 model selected by ModelFinder. Black circles represent bootstrap support ≥70% of 1000 ultrafast bootstrap replicates. MAGs obtained in this study are shown in red. On the right, average nucleotide identities (ANI) and average amino acid identities (AAI) are shown. The lower right part of the heatmap shows AAI values, the upper left part shows ANI values ≥70%; ANI values <70% are replaced by white squares. On the right side of the heatmap species classifications according to GTDB-Tk r214 are shown.

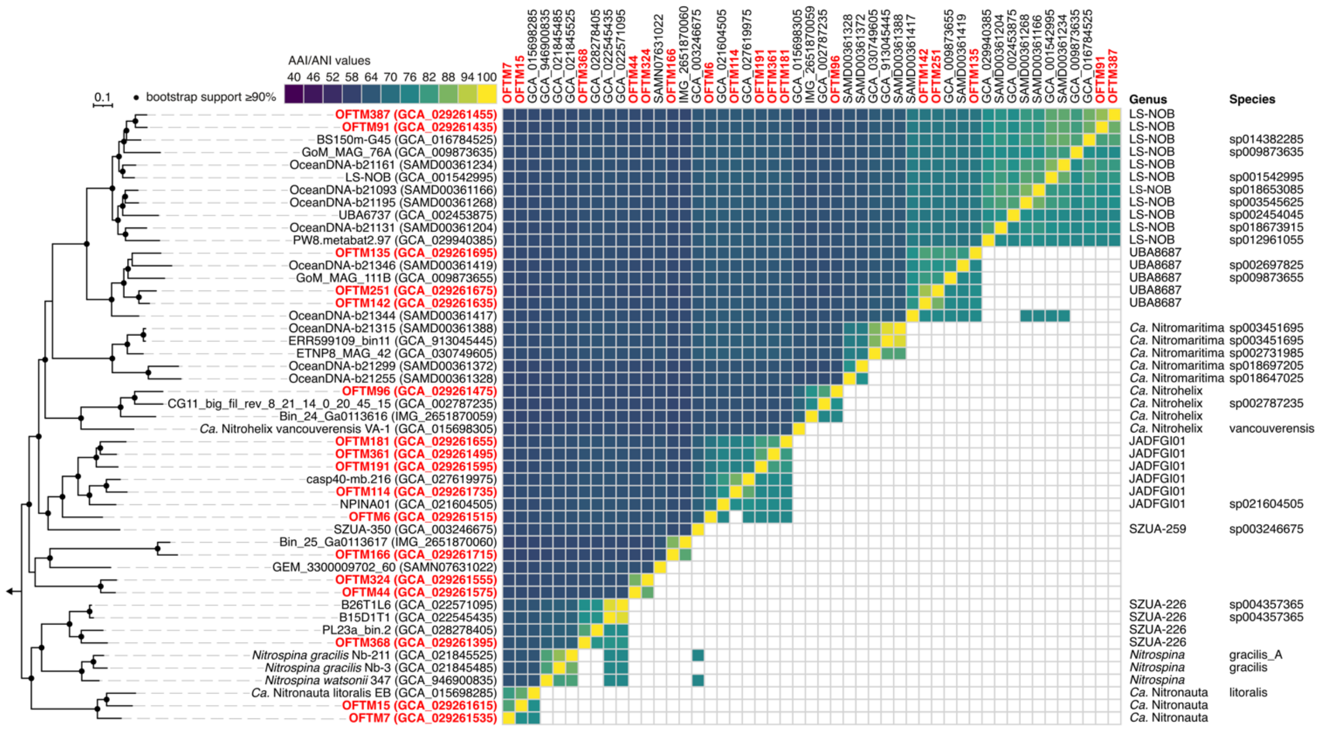

Figure S7. On the left, a phylogenomic tree of dereplicated high quality (≥90% completeness, ≤5% redundancy) family *Nitrospinia* genomes based on concatenated alignments of 92 core protein sequences is shown. The maximum likelihood tree was calculated using IQ-tree with the SYM+I+G4 model selected by ModelFinder. Black circles represent bootstrap support ≥70% of 1000 ultrafast bootstrap replicates. MAGs obtained in this study are shown in red. On the right, average nucleotide identities (ANI) and average amino acid identities (AAI) are shown. The lower right part of the heatmap shows AAI values, the upper left part shows ANI values ≥70%; ANI values <70% are replaced by white squares. On the right side of the heatmap genus and species classifications according to GTDB-Tk r214 are shown.

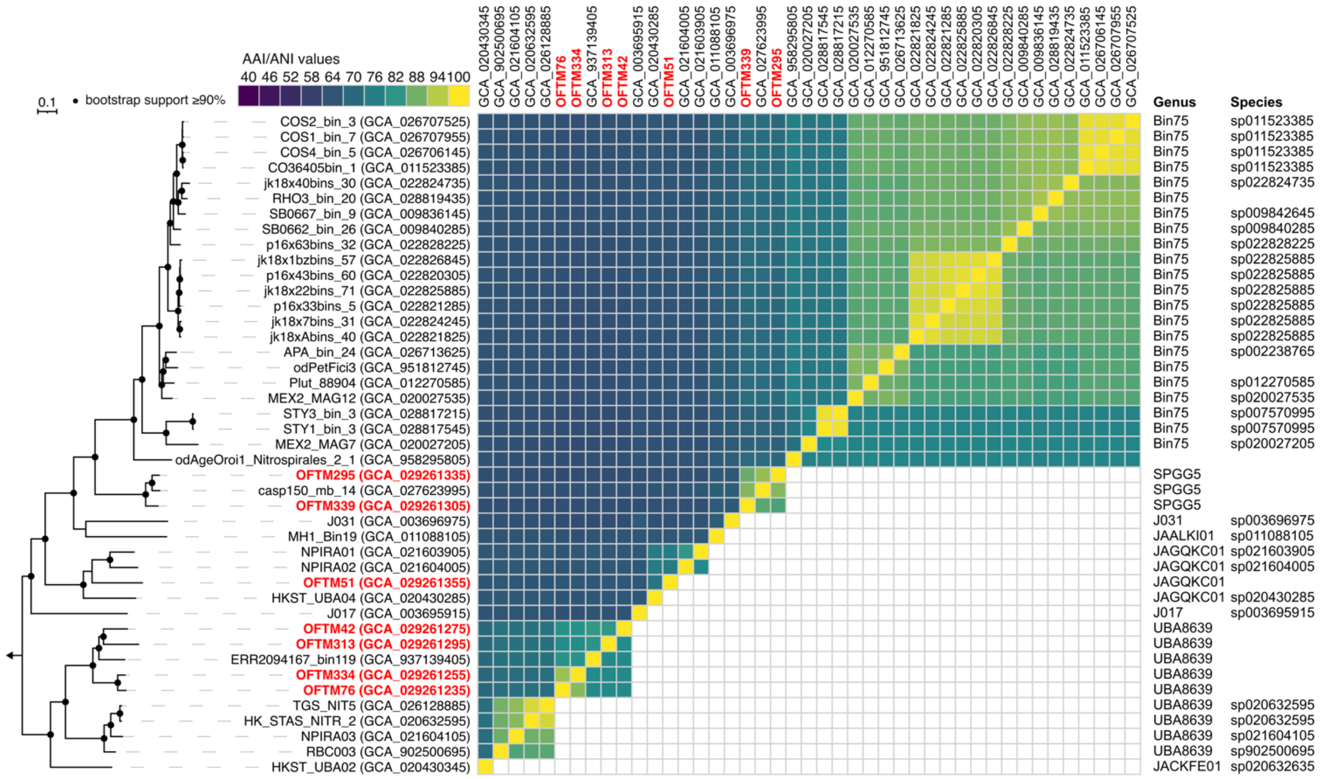

Figure S8. On the left, a phylogenomic tree of dereplicated high quality (≥90% completeness, ≤5% redundancy) family UBA8639 (order *Nitrospirales*) genomes based on concatenated alignments of 92 core protein sequences is shown. The maximum likelihood tree was calculated using IQ-tree with the GTR+F+I+G4 model selected by ModelFinder. Black circles represent bootstrap support ≥70% of 1000 ultrafast bootstrap replicates. MAGs obtained in this study are shown in red. On the right, average nucleotide identities (ANI) and average amino acid identities (AAI) are shown. The lower right part of the heatmap shows AAI values, the upper left part shows ANI values ≥70%; ANI values <70% are replaced by white squares. On the right side of the heatmap genus and species classifications according to GTDB-Tk r214 are shown.

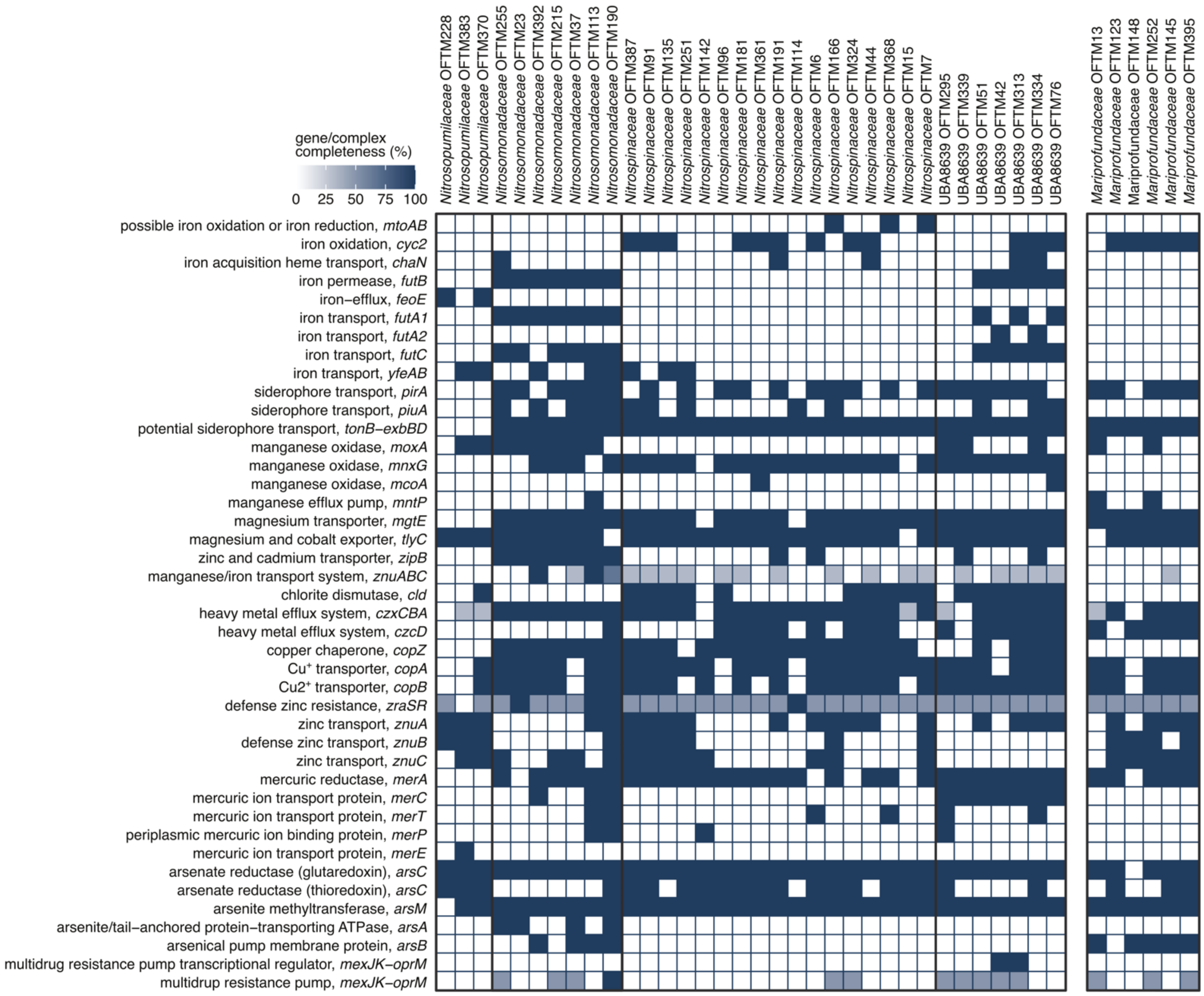

Figure S9. Heatmap showing the presence and completeness of genes and pathways involved in metal and metalloid metabolism for MAGs belonging to the families *Nitrosomonadaceae*, *Nitrosopumilaceae*, *Nitrospinaceae*, UBA8639 (order *Nitrospirales*), and *Mariprofundaceae.*

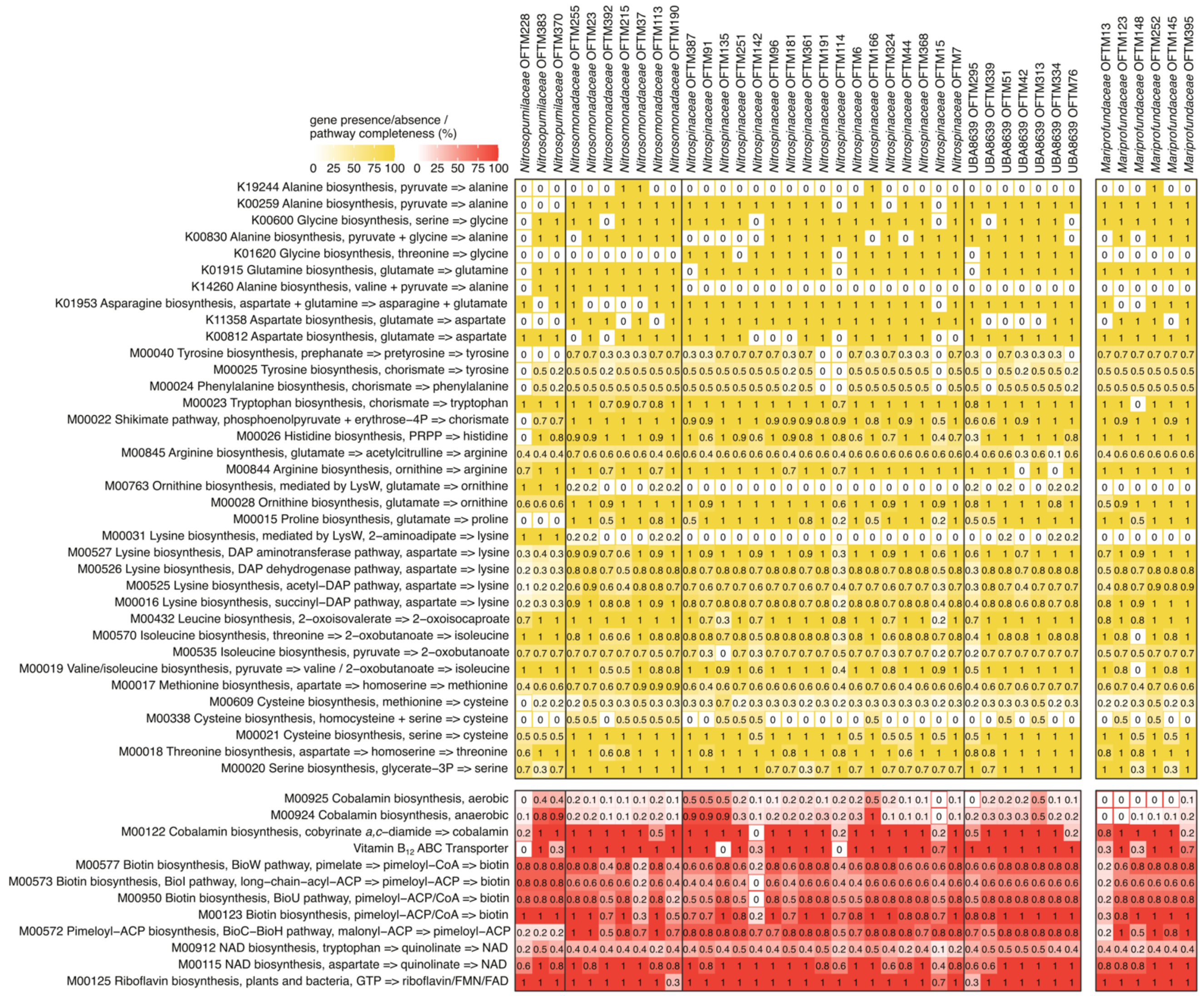

Figure S10. Heatmap showing the presence and completeness of genes and pathways involved in amino acid biosynthesis and vitamin B_{12} metabolism for MAGs belonging to the families *Nitrosomonadaceae*, *Nitrosopumilaceae*, *Nitrospinaceae*, UBA8639 (order *Nitrospirales*), and *Mariprofundaceae.* The numbers indicate the calculated pathway completeness.
